# A state model of transcription and co-transcriptional processing enables measurement of the underlying kinetics

**DOI:** 10.21203/rs.3.rs-10830564/v1

**Published:** 2026-10-19

**Authors:** Matthew Gamble, Joshua Saltzberg, Alyssa Fronk, Charles Query, Kenny Ye

## Abstract

The production of mature mRNA isoforms is governed by the kinetics of transcription and co-transcriptional RNA processing, yet these processes remain difficult to quantify simultaneously. Here, we introduce SKaTER, a mathematical state model of transcription that jointly infers the kinetics of elongation, splicing, and cleavage from either DRB released nascent RNA sequencing or a combination of PRO-seq and TT-seq. Rate measurements are reproducible and highly concordant across cell lines. Cleavage rates are strongly associated with the presence of the canonical polyadenylation signals and can be impacted by RNA binding proteins such as SRSF9. Splicing rates are dominated by spatial factors, with neighboring introns and introns in the same TAD showing strong correlations. Elongation rates are heavily determined by local chromatin marks, with rates varying both with intensity and combinations of different marks. Our framework enables joint kinetic analysis of mRNA biogenesis revealing how sequence, nuclear organization and chromatin state shape co-transcriptional RNA processing.

